# Neighbourhood social gifting and multiple long-term conditions: a nationally representative analysis of the Scottish population aged 40–75 years

**DOI:** 10.1093/eurpub/ckaf238

**Published:** 2026-02-16

**Authors:** Chunyu Zheng, Eleojo Abubakar, Katherine Keenan, Kathryn Halliday, Chris Dibben, Bruce Guthrie, Alan Marshall, Jamie Pearce

**Affiliations:** Centre for Research on Environment, Society and Health (CRESH), School of GeoSciences, University of Edinburgh, Edinburgh, United Kingdom; Institute of Population Health, Faculty of Health and Life Sciences, University of Liverpool, Liverpool, United Kingdom; School of Geography and Sustainable Development, University of St Andrews, St Andrews, United Kingdom; University of Oxford, Oxford, United Kingdom; School of GeoSciences, University of Edinburgh, Edinburgh, United Kingdom; Advanced Care Research Centre, University of Edinburgh, Edinburgh, United Kingdom; Usher Institute, College of Medicine and Veterinary Medicine, University of Edinburgh, Edinburgh, United Kingdom; School of Social and Political Science, University of Edinburgh, Edinburgh, United Kingdom; Centre for Research on Environment, Society and Health (CRESH), School of GeoSciences, University of Edinburgh, Edinburgh, United Kingdom

## Abstract

Little is known regarding the relationship between the local social environment and multiple long-term conditions (MLTC, also referred to as multimorbidity). We investigated the association between social gifting, the neighbourhood-level latent willingness to gift time for community reciprocity, and four measures of MLTC presence (‘2+ long-term conditions (LTCs)’, mental-physical MLTC, ‘3+ LTCs’ and complex MLTC). We further explored variations in these relationships across types of urban–rural settlement. We linked participants of the Scottish Longitudinal Study who participated in Census 2011, aged 40–75, with no MLTC before 2010 (*n* = 98 296), to their hospitalisation records (2010–19) and an established neighbourhood-level index reflecting social gifting. Two-level logistic regression was used to model the onset of MLTC (2010–19), accounting for the clustered data structure of individuals nested within neighbourhoods. Lower social gifting was associated with increased odds of MLTC in all measures, except for ‘2+ LTCs’, with the strongest association observed for mental-physical MLTC. There was a statistically significant interaction between social gifting and types of urban–rural settlement for mental-physical MLTC but not for other measures of MLTC, suggesting that social gifting was more strongly associated with mental-physical MLTC in urban than other areas. The findings highlight the important role of the local social environment in the development of MLTC. Policies targeted at supporting neighbourhood-level social cohesion and social participation may benefit population health, particularly for mental-physical MLTC in urban areas where observed associations were strongest.

## Introduction

The rise in people living with multiple long-term conditions (MLTC) has emerged as a global public health challenge [1]. MLTC is associated with reduced quality of life, and increased reliance on health and social care services, leading to greater health service costs and significant social and political challenges [2, 3] The prevalence of MLTC is socially patterned, with individuals living in the most disadvantaged areas developing MLTC 10–15 years earlier than those living in the most affluent areas [2], and these social differences are widening [4, 5]. Therefore, there is an urgent need to better understand the mechanisms underlying the development of MLTC to inform policy interventions.

Individual (e.g. age, sex, lower socioeconomic status (SES), and poor health behaviours) [6, 7] and household-level characteristics (e.g. household income, household composition, and household tenure) have been found to be associated with MLTC [8]. Additionally, features of the places where people reside may affect MLTC, individuals living in more deprived, more urbanised, or highly polluted areas are more likely to have MLTC, although results can be sensitive to the operationalisation of MLTC measures [9]. However, our understanding of how other social characteristics of place affect MLTC remains limited.

Neighbourhood social cohesion relates to the positive dimensions of the local social environment, such as trust, sense of belonging, and willingness to participate and help [10]. Research suggests that living in more socially cohesive neighbourhoods has improved health outcomes [11–14]. A key dimension of social cohesion is social gifting, which refers to people’s willingness to donate their time to others (i.e. voluntary social participation) [15]. Social gifting can lead to meaningful benefits to wellbeing [16], mental health [17], and physical health [18]. Social gifting has been linked to mental health conditions in Scotland by using a newly developed neighbourhood-level index—the Social Gifting Index (SGI). Residing in areas with lower social gifting is associated with an increased likelihood of having mental health conditions after accounting for individual- and area-level covariates, with significant differences between living in remote islands and other areas [15, 17]. Given that MLTC are a complex combination of health conditions, it is plausible that social gifting may also be associated with MLTC. Here we extend the literature on social gifting to see whether social gifting is associated with MLTC and to evaluate whether it is meaningful across Scotland more generally, including outside remote island communities.

A few studies have explored how social cohesion [19], including its dimensions such as social participation [20, 21] social support [22], and social isolation [21, 23] relates to MLTC, with findings being equivocal. Potential reasons for this discrepancy are twofold. First, the measures reflecting social cohesion varied greatly across studies and were mostly operationalised as individual attributes rather than capturing contextual characteristics relating to people’s local environment [24]. Second, the measurement of MLTC varies between studies in terms of both how data is collected (self-report vs. medical records) and which conditions are counted [9, 25, 26] There is a need for new work operationalising neighbourhood social cohesion, linking multiple databases at a national scale, utilising medical records, and applying different MLTC measurements. Therefore, this study examines the relationship between social gifting and MLTC under different measurements using nationally representative data that links the population census and secondary-care database of Scotland over the period 2010–19 with an established neighbourhood-level index that reflects social gifting and explores whether this relationship varies by types of urban–rural settlement.

## Methods

### Study sample

The study sample was drawn from the Scottish Longitudinal Study (SLS), a 5.3% representative sample of the Scottish population followed across three censuses (1991, 2001, and 2011), and linked with inpatient hospitalisation records (SMR01 and SMR04, 1 January 1997–31 August 2019) and the cancer registry (SMR06, 1 January 1980–31 August 2019). The analytical samples were restricted to individuals present at the 2011 census, aged 40–75 years old during the enumeration. To minimise reverse causation—whether lower social gifting leads to higher MLTC risks, or higher MLTC prevalence within neighbourhoods reduces social gifting—SLS members diagnosed with MLTC before January 2010, the beginning of the study period, were excluded (Supplementary Fig. S1).

### Outcome

Our outcome of interest, MLTC, has varying measurements and definitions [25]. We extracted the presence of LTCs based on ICD-10 codes using a recently developed consensus list of 47 LTCs to include in MLTC measurement for research (Supplementary Table S1) [27]. The presence of a condition in an individual was recorded based on its earliest date. The outcome was the development of MLTC during the follow-up period from January 2010 to August 2019, using four definitions of MLTC (all binary outcomes, used in separate analyses): (i) 2+ LTCs: co-occurrence of any 2 or more conditions; (ii) Mental-physical MLTC: co-occurrence of at least one mental condition and at least one physical condition [2, 25, 28]; (iii) 3+ LTCs: co-occurrence of any 3 or more conditions; (iv) Complex MLTC: co-occurrence of any 3 or more conditions from 3 or more body systems [25, 28] SLS members with the first occurrence of these four measures during follow-up were coded as ‘1’ respectively, while those with no hospitalisation records, no indexed conditions, or only one indexed condition throughout the period were coded as ‘0’.

### Exposure

The exposure of interest is social gifting, measured by the SGI, which quantifies the neighbourhood social voluntary participation. The SGI was developed by pooling response rates from all main Scottish Government surveys (i.e. the Scottish Health Survey, the Scottish Household Survey, and the Scottish Crime and Justice Survey) in 2012–17 [15, 17]. To derive stable estimates of the datazone-level refusal rate, a multilevel analytical framework (datazones nested in intermediate zones nested in local authorities) amongst the eligible population (sample frame and present at residence) was used with shrinkage modelling [15, 17]. Datazone-level refusal rates are the weighted and summed percentages that reflect local residents’ latent willingness to voluntarily participate in national surveys that are relevant to public interests regarding citizens’ wellbeing, household composition, public experiences, and perceptions of crime, so that the highest refusal at the datazone level reflects the lowest social gifting [15]. To more accurately capture how social gifting is related to MLTC throughout the full range of the index across Scotland and to follow the measure being used in the previous study [17], SGI was fitted as a continuous exposure, where the higher the value of the index, the lower the level of social gifting in neighbourhoods. Sensitivity tests using different measures of SGI (i.e. continuous exposure of SGI vs. quintiles of SGI) were appended to the supplementary materials, with substantive conclusions unaffected (Supplementary Tables S2–S10).

### Covariates

Derived from the 2011 Census, the Scottish Index of Multiple Deprivation (SIMD 2012), and the Scottish Government Urban Rural Classification (2013–14), individual demographics (age, sex, and ethnic group), SES (marital status, highest level of educational qualifications, social grade, and economic activity), household-level SES (household tenure), as well as area-level characteristics (area-level income deprivation and types of urban–rural settlement) were included as covariates (see Supplementary Table S11 for details). As the covariates’ missing rate was <3%, all missing values were removed for complete case analysis.

### Statistical analysis

To account for the clustered structure of multiple administrative data, with individuals (level 1) clustered within datazones (level 2, average population 500–1000, akin to neighbourhood), two-level logistic regression was applied to analyse the association between social gifting and MLTC. To examine the main effect, individual- and household-level socio-demographics, area-level income deprivation, and three-fold urban–rural classification were controlled. Sensitivity analyses with varying covariate adjustments were also performed (Supplementary Fig. S2 and Table S12). Further, we tested the interaction between social gifting and types of urban–rural settlement (SGI × 3-level urban–rural settlements). Each set of models was conducted for four different MLTC measurements. The intra-class correlation (ICC) and Akaike Information Criterion (AIC) were reported along with the model outputs. The Wald test was applied to measure whether the interaction effect of types of urban–rural settlement is statistically significant [29]. Adjusted predictions at the representative values were calculated and plotted to visualise the interaction effect. Statistical significance was defined at P < .05. All analyses were performed using STATA 16.1 MP, with data visualisation carried out in R using the ggplot2 package.

### Ethics

The ethics clearance was received from the School of GeoSciences Research Ethics & Integrity Committee, the University of Edinburgh (reference: 2023-703). This study was also approved by the SLS Research Board (SLS project number 2018_012) and by the Public Benefit and Privacy Panel for Health and Social Care of NHS Scotland (reference: 1819–0093). All analyses were performed in accordance with the relevant SLS guidelines and regulations.

## Results

The analyses included 98 296 SLS members aged 40–75 at the 2011 Census, who were without MLTC before 2010 (Table 1). Across all analytical samples, the mean age was 55.6 (SD = 9.6) years, 52.8% were female, 98.6% were white, two-thirds were married (65.5%), 30.6% had no educational qualifications, 64.2% were employed, one-third (33.6%) were amongst the lowest tier of social grade (class ‘DE’), 78.2% owned properties, 66.8% resided in urban areas, and 23.2% resided in the least deprived neighbourhoods (Q5). Throughout the study period (2010–19), 18.9% of the analytical samples had their first occurrence of ‘2+ LTCs’, compared to 3.5% for ‘mental-physical MLTC’, 10.0% for ‘3+ LTCs’ and 7.0% for ‘complex MLTC’. The SGI ranges from 11.22–34.51 across Scotland, with one unit of the index values representing the partially pooled percentage of refusal within the neighbourhood.

**Table 1.** Sample characteristics (*source*: Scottish Longitudinal Study).

	Characteristics	
All (*n*)	98 296	
‘2+ LTCs’ (*n*, %)	18 524	18.85
‘Mental-physical MLTC’ (*n*, %)	3396	3.45
‘3+ LTCs’ (*n*, %)	9862	10.03
‘Complex MLTC’ (*n*, %)	6850	6.97
Age based on census 2011 (mean, SD)	55.55	9.56
Age in 10-year group based on census 2011 (*n*, %)		
40–49	32 038	32.59
50–59	31 448	31.99
60–69	25 099	25.53
70+	9711	9.88
Sex (*n*, %)		
Male	46 445	47.25
Female	51 851	52.75
Ethnic group (*n*, %)		
White	96 920	98.60
Other ethnic groups	1376	1.40
Highest-level educational qualifications (*n*, %)		
No qualifications	30 070	30.59
Low	21 728	22.10
Medium	20 569	20.93
High	25 929	26.38
Economic activity (*n*, %)		
Employed	63 090	64.18
Retired	23 315	23.72
Not in the labour force	9111	9.27
Unemployed	2780	2.83
Social grade (*n*, %)		
AB	24 894	25.33
C1	25 442	25.88
C2	14 967	15.23
DE	32 993	33.56
Marital status (*n*, %)		
Single	12 606	12.82
Married	64 390	65.51
Divorced, separated, widowed	21 300	21.67
Household tenure (*n*, %)		
Owned	76 843	78.18
Privately rented	4389	4.47
Socially rented	16 310	16.59
Live rent free	754	0.77
Area-level income deprivation (*n*, %)		
Q1 (most deprived)	15 747	16.02
Q2	18 125	18.44
Q3	19 586	19.93
Q4	22 082	22.46
Q5 (least deprived)	22 756	23.15
Urban–rural settlements (*n*, %)		
Urban areas	65 690	66.83
Small towns	13 280	13.51
Rural areas	19 326	19.66

**Figure 1.**
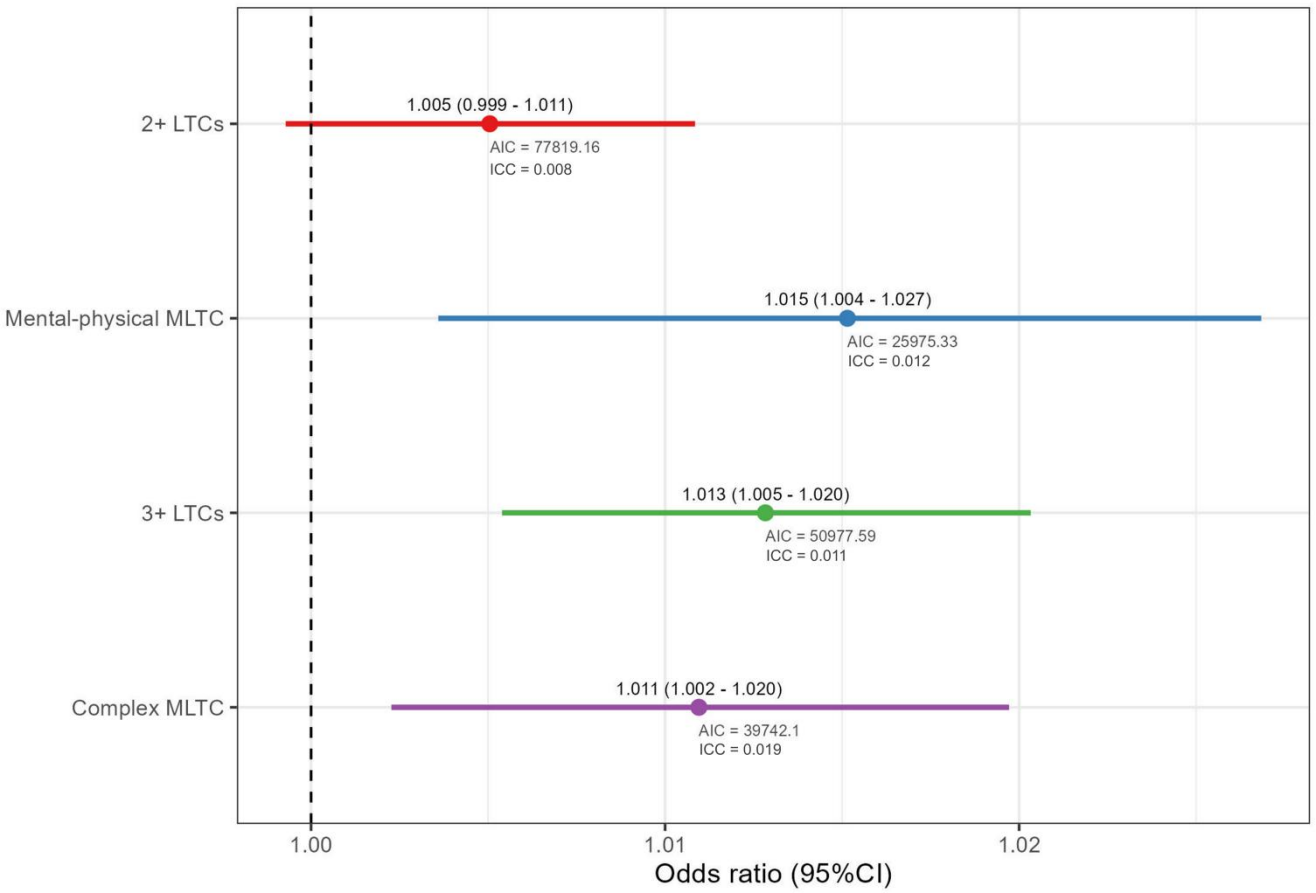
The main effect of social gifting on MLTC using four different definitions (*source*: Scottish Longitudinal Study).

Figure 1 presents the association between social gifting and MLTC with the adjustment for individual, household, and area-level covariates, but without any interaction terms fitted. There is little evidence for the association between social gifting and ‘2+ LTCs’ (*P* values = .086). Unlike ‘2+ LTCs’, higher values of the index (i.e. lower social gifting) were found to be significantly associated with an increased likelihood of having MLTC under other measures, with the strongest association being observed for mental-physical MLTC (odds ratio [OR] = 1.015, 95% confidence interval [CI] = 1.004, 1.027), indicating that there was a 1.5% increase in the odds of having mental-physical MLTC for each 1% increase in the neighbourhood-level partially pooled percentage of refusal, followed by ‘3+ LTCs’ (OR = 1.013, 95% CI = 1.005, 1.020) and complex MLTC (OR = 1.011, 95% CI = 1.002, 1.020).

**Figure 2.**
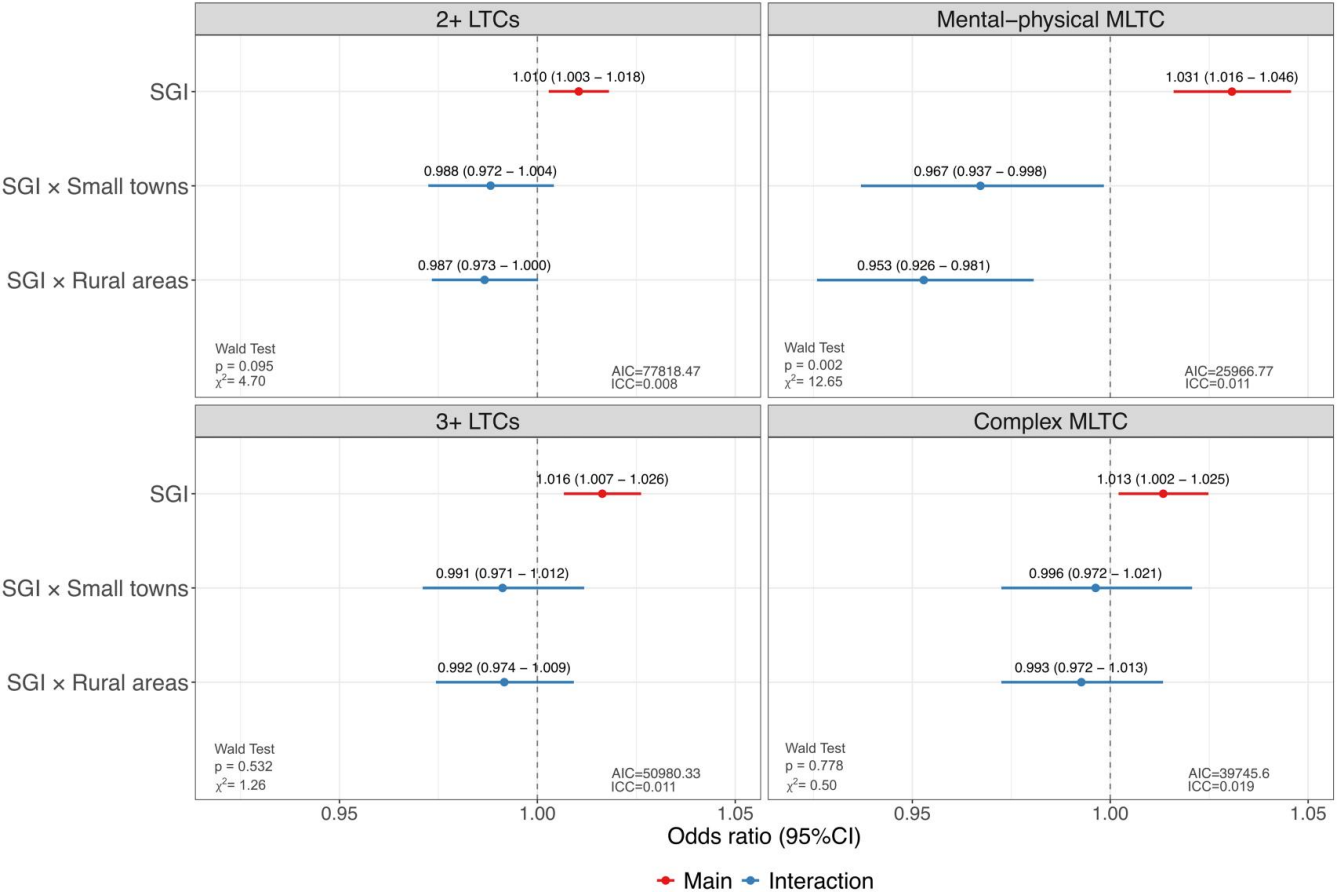
Interactions between social gifting and types of urban–rural settlement (*source*: Scottish Longitudinal Study).

**Figure 3.**
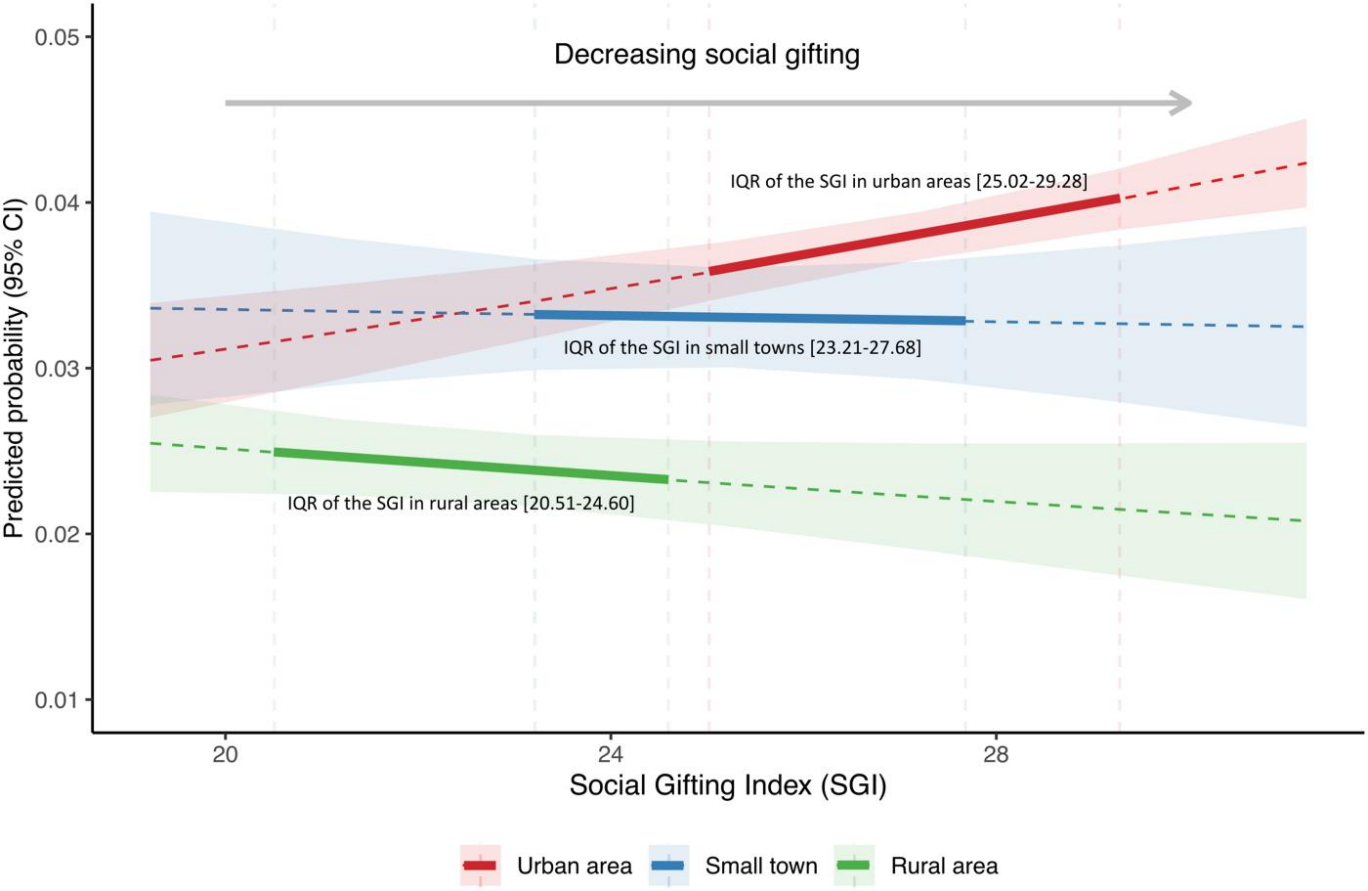
The predicted probability of mental-physical MLTC by social gifting across types of urban–rural settlement (*source*: Scottish Longitudinal Study).

Figure 2 presents the interaction effect between SGI and types of urban–rural settlement using different measures of MLTC. After including the interaction terms between social gifting and types of urban–rural settlement, for all measurements of MLTC, the main effect of SGI suggested that the positive association between higher values of the index (i.e. lower social gifting) and the increased ORs of MLTC in urban areas, with a reversed trend being found in small towns and rural areas. The Wald test suggested that the interaction effect between SGI and types of urban–rural settlement was statistically significant for mental-physical MLTC only. Therefore, Fig. 3 focuses on mental-physical MLTC by presenting adjusted predictions illustrating the interaction between social gifting and types of urban–rural settlement. Predicted probabilities are shown across the interquartile range (IQR) of SGI for each type of urban–rural settlement. Rural areas (IQR of SGI: 20.51–24.60) and small towns (IQR of SGI: 23.21–27.68), characterised by higher social gifting than urban areas (IQR of SGI: 25.02–29.28), showed overall lower predicted probabilities of mental-physical MLTC. We also observed that there were increases in probabilities of mental-physical MLTC as social gifting worsened (increasing values of the index) in urban areas, while the association was reversed in small towns and rural areas. Nevertheless, the slope in the predicted probability of mental-physical MLTC with changes in the values of SGI was essentially flat in small towns and rural areas. The rate of change in the predicted probability of having mental-physical MLTC per unit change in the values of SGI was tenfold greater in urban areas as compared to small towns, and it was approximately twice as great as in rural areas.

## Discussion

Using a nationally representative linked dataset from Scotland, we found that residents in areas with lower social gifting were more likely to develop MLTC. The strongest association was found for mental-physical MLTC, largely driven by the associations in urban areas. In contrast, small towns and rural areas, characterised by lower probabilities of MLTC and stronger levels of social gifting, exhibited a weaker and reversed pattern.

We observed the association between lower social gifting and increased likelihood of MLTC in most measurements, but with the strongest association being observed for mental-physical MLTC. One possible explanation is that social gifting tends to be more directly associated with mental health conditions than physical conditions. Halliday, et al. [17] observed the link between SGI and mental health, measured by either self-reported mental illness or whether receiving prescriptions for antidepressant and/or anxiolytic medications. While Kim, et al. [13] and Lee, et al. [30] found that perceived social cohesion was more strongly associated with mental wellbeing and distress outcomes rather than physical conditions [13, 30] Although mental health measurements differ across studies (i.e. self-reported status/illness vs. primary-care prescription vs. secondary-care diagnosis), these aligned findings may imply the more direct benefits of social gifting on mental health. Areas with more residents willing to gift time for community reciprocity may have increased social trust, strengthened bonding ties, and reduced social exclusions, which may deliver positive benefits for mental health and wellbeing that are protective against the development of other mental problems [15]. While the reduced incidence of mental health conditions may further contribute to the lower risk of physical conditions as well as mental-physical MLTC. Several prior works have indicated that people living with mental health conditions may have a higher prevalence of physical conditions [31, 32] with growing evidence further suggesting increased MLTC risks among people with severe mental health conditions [33].

Our interaction analyses further suggested that there may be differences in how social gifting was associated with MLTC by types of urban–rural settlement. The link between social gifting and MLTC was mostly driven by the patterns in urban areas, which accounted for over 60% of the total analytical samples. Unlike the pattern in urban areas, lower social gifting was associated with reduced mental-physical MLTC risks in non-urban areas, but the strength of the association was weak given the notably flatter slope for small towns and rural areas. This finding does not necessarily imply that social gifting negatively affects mental-physical MLTC in non-urban areas. Our exposure, the SGI, is unevenly distributed across types of urban–rural settlement, with the values more dispersed in non-urban areas as compared to those in urban areas (Supplementary Fig. S3). It is possible that there is a threshold of ‘poor social gifting’ before an association is observed between SGI and MLTC, while small towns and rural areas do not sufficiently cross into the threshold of ‘poor social gifting’ for us to observe the association with mental-physical MLTC. In addition to the uneven distribution of the SGI, the observed interaction pattern may reflect the limited variation regarding this link in non-urban areas due to the small number of communities. Moreover, our MTLC measures were ascertained from hospitalisation records, where known variation in hospital admissions by distance may contribute to urban–rural differences in hospital admissions [34] for mental conditions [35] or mental-physical MLTC. Combined with differences in social support and available resources at the local level, this may lead to heterogeneity in the association between social gifting and mental-physical MLTC through differing pathways linking exposure and outcome.

This is the first study to consider the association between a key component of the local social environment and various MLTC measures at a national scale. The large-scale data linkage between multiple databases, including administrative data (i.e. population census, Scottish Government surveys) and hospitalisation records, enables the integration of individual-, household-, and area-level characteristics from a more geographically representative analytical sample, allowing us to provide a more robust estimation. However, some limitations need to be noted. First, although we tried to eliminate the influence of reverse causation by restricting our analytical samples to SLS members with no MLTC at the baseline, reverse causality remains a possibility. As our MLTC measures were derived from secondary-care data, it is possible that our analytical sample included SLS members who were either diagnosed with MLTC in 2010–12 or prior to 2010 in primary-care settings but had not been hospitalised for MLTC throughout the analysis period. Also, since our hospitalisation records do not cover the full life course of the SLS members, there might be a chance that SLS members were hospitalised for any included conditions prior to 2001, for which we cannot trace it back (SMR01 and SMR04, traceable back to January 1997 but with more complete records from 2001 onwards) unless it was cancer (derived from SMR06, traceable back to January 1980). As a result, examining the effect of social gifting might still be affected by these subsamples. Future research could consider using causal inference methods (e.g. instrumental variables) to further determine the direction of the pathway linking social gifting and MLTC. Second, the absence of primary-care data and by relying on hospitalisation records may underestimate the incidence of certain LTCs, such as mental and behavioural and sensory disorders, as compared to using primary care data [27]. This omission may misrepresent differences between people living with mild MLTC and those who were hospitalised, particularly among people with mental–physical MLTC. However, data linkage between the Scottish population census and primary care data at the national scale is not yet available. Future research should consider utilising both primary- and secondary-care records when data are available. Third, although we followed up SLS members with no MLTC for almost 10 years, as the linkage between SGI and the census was based on residential address at the time of enumeration, any residential moves occurring after the census could not be accounted for in our analysis. Future research should consider using residential history data to further explore how changing social environments may affect MLTC. Fourth, it is plausible that local residents are eager to gift their own time to neighbourhood mutual benefits but may be unwilling to give time for the national surveys conducted by the Scottish Government. Nevertheless, the SGI has been proven to have the capacity to capture some contextual features of neighbourhoods related to social cohesion that go beyond the Social Fragmentation Index (another widely used measure for social cohesion) [17]. Lastly, although all adjusted models have controlled for individual-, household-, and area-level covariates, residual confounding (e.g. other components of the local social environment) remains a possibility. The local social environment involves the interplay between multiple components, including social gifting. When data is available, it is beneficial to further explore how these components (e.g. ethnic diversity, social integration, community-level incivility) may influence MLTC individually or collectively, which may enhance our understanding of the mechanism behind how local social environments affect health.

This study provides evidence regarding the link between social gifting, a key component of the local social environment, and measures of MLTC across Scotland and how these relationships vary across types of urban–rural settlement. The findings help elucidate the mechanism regarding how social characteristics of ‘place’ affect the development of MLTC and indicate that social gifting may play a role in the development of MLTC. Our findings have policy implications that initiatives to support social cohesion and participation may form part of a strategy to address the rapid increase in MLTC prevalence, although these initiatives are likely to be more effective for mental-physical MLTC or in urban areas. It has been suggested that cuts to local government service expenditure may adversely influence local-level social participation by directly affecting the public services and resources, especially for the more disadvantaged individuals or communities [36], while cuts to local government service expenditure were found to be associated with the increasing MLTC prevalence [37]. Therefore, it is possible that initiatives such as investment in local government services might help create a more cohesive social environment that encourages more voluntary participation, which has the potential to intervene in the rapid growth of MLTC prevalence and reduce health inequalities.

## Supplementary Material

ckaf238_Supplementary_Data

## Data Availability

The data underlying this article were provided by the SLS (https://sls.lscs.ac.uk) under licence. Restrictions apply and they are not publicly available. Key points Lower social gifting was associated with higher risks of MLTC in all measures but ‘2+ LTCs’. A stronger association was found for mental-physical MLTC than for other MLTC measures. The association with mental-physical MLTC differed statistically by urban–rural settings. The overall association with mental-physical MLTC was mostly driven by that in urban areas. Policies promoting local cohesion may help reduce the rise of MLTC, particularly for mental-physical MLTC or in urban areas.
