# Supplementary material for: Neighbourhood social gifting and multiple long-term conditions: a nationally representative analysis of the Scottish population aged 40–75 years
Source: Eur J Public Health. 2026 Feb 16;36(2):ckaf238. doi: 10.1093/eurpub/ckaf238 (PMC13017652; doi:10.1093/eurpub/ckaf238)
Supplement: ckaf238_Supplementary_Data [file ckaf238_supplementary_data.zip › 07-Feb-2026_010856_ejph-2025-09-om-0762-File005.docx]

**Supplementary Material**

Contents

[**1. Research timeline and data linkage** 2](#_Toc215139130)

[**Figure S1.** Timeline and data linkage 2](#_Toc215139131)

[**2. List of 47 long-term conditions** 3](#_Toc215139132)

[**Table S1.** List of 47 long-term conditions 3](#_Toc215139133)

[**3. Sensitivity tests on different formats of the SGI** 5](#_Toc215139134)

[**Table S2.** Exposure formats 5](#_Toc215139135)

[**Table S3.** Regression outputs for ‘2+ LTCs’ (source: Scottish Longitudinal Study) 5](#_Toc215139136)

[**Table S4.** Likelihood Ratio Test for ‘2+ LTCs’ (source: Scottish Longitudinal Study) 5](#_Toc215139137)

[**Table S5.** Regression outputs for ‘mental-physical MLTC’ (source: Scottish Longitudinal Study) 5](#_Toc215139138)

[**Table S6.** Likelihood Ratio Test for ‘mental-physical MLTC’ (source: Scottish Longitudinal Study) 5](#_Toc215139139)

[**Table S7.** Regression outputs for ‘3+ LTCs’ (source: Scottish Longitudinal Study) 5](#_Toc215139140)

[**Table S8.** Likelihood Ratio Test for ‘3+ LTCs’ (source: Scottish Longitudinal Study) 6](#_Toc215139141)

[**Table S9.** Regression outputs for ‘complex MLTC’ (source: Scottish Longitudinal Study) 6](#_Toc215139142)

[**Table S10.** Likelihood Ratio Test for ‘complex MLTC’ (source: Scottish Longitudinal Study) 6](#_Toc215139143)

[**4. Description of the covariates** 7](#_Toc215139144)

[**Table S11.** Description of covariates (source: Scottish Longitudinal Study) 7](#_Toc215139145)

[**5.** **Sensitivity test for models with varying adjustments** 9](#_Toc215139146)

[**Figure S2.**  The main effect of SGI on MLTCs under four different measurements (source: Scottish Longitudinal Study) 9](#_Toc215139147)

[**Table S12**. Regression outputs from the main effect of SGI (source: Scottish Longitudinal Study) 10](#_Toc215139148)

[**6. Distribution of the SGI across types of urban-rural settlement** 11](#_Toc215139149)

[**Figure S3**. The Distribution of SGI across types of urban-rural settlement 11](#_Toc215139150)

# **1. Research timeline and data linkage**


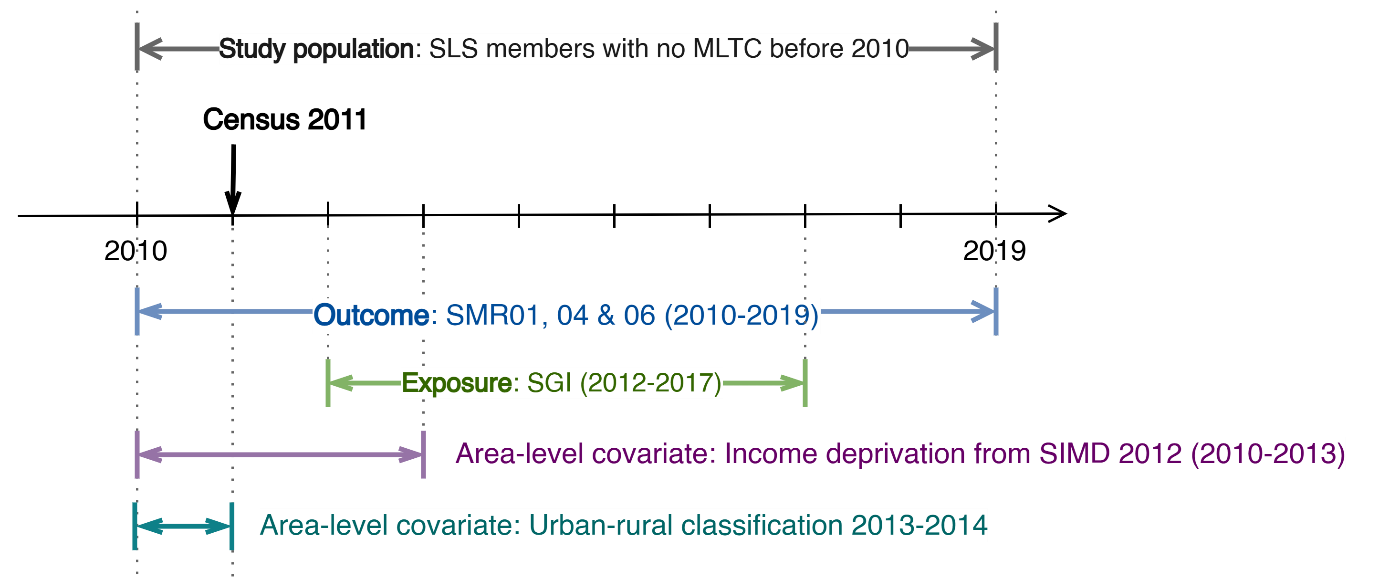


## **Figure S1. Timeline and data linkage**

# **2. List of 47 long-term conditions**

For creating the measures for multiple long-term conditions (MLTC), we followed a list of 47 long-term conditions,^1^ the choice of the 47 long-term conditions was based on results of a recent Delphi consensus study.^2^

## **Table S1.** List of 47 long-term conditions

| **ICD-10 body system** | **Long-term condition** |
| --- | --- |
| I - Certain infectious and parasitic diseases | Tuberculosis |
| II - Neoplasms | Cancer |
| III - Diseases of blood/blood-forming organs | Anaemia |
| IV - Endocrine, nutritional and metabolic diseases | Cystic fibrosis |
| IV - Endocrine, nutritional and metabolic diseases | Thyroid disorders |
| IV - Endocrine, nutritional and metabolic diseases | Addison's disease |
| IV - Endocrine, nutritional and metabolic diseases | Diabetes |
| V - Mental and behavioural disorders | Post traumatic stress disorder |
| V - Mental and behavioural disorders | Depression |
| V - Mental and behavioural disorders | Eating disorder |
| V - Mental and behavioural disorders | Anxiety |
| V - Mental and behavioural disorders | Autism |
| V - Mental and behavioural disorders | Alcohol and substance misuse |
| V - Mental and behavioural disorders | Bipolar affective disorder |
| V - Mental and behavioural disorders | Dementia |
| V - Mental and behavioural disorders | Schizophrenia |
| VI - Diseases of the nervous system | Peripheral neuropathy |
| VI - Diseases of the nervous system | Parkinson's disease |
| VI - Diseases of the nervous system | Multiple sclerosis |
| VI - Diseases of the nervous system | Epilepsy |
| VI - Diseases of the nervous system | Paralysis |
| VII - Diseases of the eye and adnexa | Visual impairment |
| VIII - Diseases of the ear and mastoid process | Hearing impairment |
| VIII - Diseases of the ear and mastoid process | Meniere's disease |
| IX - Diseases of the circulatory system | Venous thromboembolism |
| IX - Diseases of the circulatory system | Peripheral artery disease |
| IX - Diseases of the circulatory system | Stroke and transient ischaemia attack |
| IX - Diseases of the circulatory system | Hypertension |
| IX - Diseases of the circulatory system | Heart failure |
| IX - Diseases of the circulatory system | Coronary artery disease |
| IX - Diseases of the circulatory system | Heart valve disorders |
| IX - Diseases of the circulatory system | Arrythmia |
| IX - Diseases of the circulatory system | Aneurysm |
| X - Diseases of the respiratory system | Bronchiectasis |
| X - Diseases of the respiratory system | Chronic obstructive pulmonary disease |
| X - Diseases of the respiratory system | Asthma |
| XIII - Diseases of the musculoskeletal system | Gout |
| XIII - Diseases of the musculoskeletal system | Connective tissue disorders |
| XIII - Diseases of the musculoskeletal system | Osteoarthritis |
| XIII - Diseases of the musculoskeletal system | Osteoporosis |
| XI – Diseases of the digestive system | Peptic ulcer |
| XI – Diseases of the digestive system | Chronic liver disease |
| XI – Diseases of the digestive system | Inflammatory bowel disease |
| XI – Diseases of the digestive system | Chronic pancreatitis |
| XIV - Diseases of the genitourinary system | Chronic kidney disease |
| XIV - Diseases of the genitourinary system | Endometriosis |
| XVII - Congenital malformations, deformations and chromosomal abnormalities | Congenital disease and chromosomal abnormalities |

**References**

1 MacRae C, Morales D, Mercer SW, et al. Impact of data source choice on multimorbidity measurement: a comparison study of 2.3 million individuals in the Welsh National Health Service. BMC Medicine 2023;21:309.

2 Ho IS-S, Azcoaga-Lorenzo A, Akbari A, et al. Measuring multimorbidity in research: Delphi consensus study. BMJ Medicine 2022;1:e000247.

# **3. Sensitivity tests on different formats of the SGI**

## **Table S2.** Exposure formats

|  | Exposure format |
| --- | --- |
| Model A | SGI |
| Model B | SGI in quintiles (Q1-Q5) |

## **Table S3.** Regression outputs for ‘2+ LTCs’ (source: Scottish Longitudinal Study)

|  |  | **OR** | **95% CI** |
| --- | --- | --- | --- |
| **Model A** | SGI | 1.005 | (0.999, 1.011) |
| **Model B** | Q1 | REF |  |
|  | Q2 | 1.080 | (1.018, 1.146) |
|  | Q3 | 1.067 | (1.003, 1.135) |
|  | Q4 | 1.058 | (0.994, 1.127) |
|  | Q5 | 1.083 | (1.016, 1.155) |

## **Table S4.** Likelihood Ratio Test for ‘2+ LTCs’ (source: Scottish Longitudinal Study)

| Assumption | Model A nested in Model B |
| --- | --- |
| $\chi^{2}$ | 5.33 |
| $p$ | 0.1489 |

## **Table S5.** Regression outputs for ‘mental-physical MLTC’ (source: Scottish Longitudinal Study)

|  |  | **OR** | **95% CI** |
| --- | --- | --- | --- |
| **Model A** | SGI | 1.015 | (1.004, 1.027) |
| **Model B** | Q1 | REF |  |
|  | Q2 | 1.034 | (0.915, 1.167) |
|  | Q3 | 1.040 | (0.918, 1.179) |
|  | Q4 | 1.146 | (1.013, 1.296) |
|  | Q5 | 1.191 | (1.051, 1.349) |

## **Table S6.** Likelihood Ratio Test for ‘mental-physical MLTC’ (source: Scottish Longitudinal Study)

| Assumption | Model A nested in Model B |
| --- | --- |
| $\chi^{2}$ | 5.10 |
| $p$ | 0.1648 |

## **Table S7.** Regression outputs for ‘3+ LTCs’ (source: Scottish Longitudinal Study)

|  |  | **OR** | **95% CI** |
| --- | --- | --- | --- |
| **Model A** | SGI | 1.013 | (1.005, 1.020) |
| **Model B** | Q1 | REF |  |
|  | Q2 | 1.057 | (0.979, 1.141) |
|  | Q3 | 1.090 | (1.006, 1.180) |
|  | Q4 | 1.096 | (1.011, 1.187) |
|  | Q5 | 1.136 | (1.047, 1.233) |

## **Table S8.** Likelihood Ratio Test for ‘3+ LTCs’ (source: Scottish Longitudinal Study)

| Assumption | Model A nested in Model B |
| --- | --- |
| $\chi^{2}$ | -1.37 |
| $p$ | 1.0000 |

## **Table S9.** Regression outputs for ‘complex MLTC’ (source: Scottish Longitudinal Study)

|  |  | **OR** | **95% CI** |
| --- | --- | --- | --- |
| **Model A** | SGI | 1.011 | (1.002, 1.020) |
| **Model B** | Q1 | REF |  |
|  | Q2 | 1.087 | (0.994, 1.189) |
|  | Q3 | 1.111 | (1.012, 1.220) |
|  | Q4 | 1.102 | (1.003, 1.211) |
|  | Q5 | 1.142 | (1.038, 1.257) |

## **Table S10.** Likelihood Ratio Test for ‘complex MLTC’ (source: Scottish Longitudinal Study)

| Assumption | Model A nested in Model B |
| --- | --- |
| $\chi^{2}$ | 2.03 |
| $p$ | 0.5654 |

**Note:** All models presented were fully adjusted models that controlled for age, sex, ethnic group, marital status, highest-level educational qualifications, social grade, economic activity, household tenure, area-level income deprivation and types of urban-rural settlement.

# **4. Description of the covariates**

## **Table S11.** Description of covariates (source: Scottish Longitudinal Study)

| **Variable** | **Description** | **Time** |
| --- | --- | --- |
| **Age** in 10-year interval (40-49/ 50-59/ 60-69/ 70+) | Based on Census 2011 for descriptive statistics.  For multilevel modelling, age was fitted using the age of onset for SLS members with MLTC records, while the rest used the census age. | Date of birth in Census 1991 and Census 2011 |
| **Sex** | | |
| Male | Based on core information of each SLS member | SLS core database |
| Female |  |  |
| **Marital status** |  |  |
| Single | Never married and never registered a same-sex civil partnership | Census 2011 |
| Married | Married or re-married; In a registered same-sex civil partnership |  |
| Divorced, separated, and widowed | Divorced; Formerly in a same-sex civil partnership which is now legally  Dissolved;  Separated, but still legally married; Separated, but still legally in a same-sex civil partnership  Widowed; Surviving partner from a same-sex civil partnership |  |
| **Highest educational qualifications** | | |
| No qualifications | No qualifications | Census 2011 |
| Low | Level 1: O Grade, Standard Grade, Access 3 Cluster, Intermediate 1 or 2, GCSE, CSE, Senior Certificate or equivalent; GSVQ Foundation or Intermediate, SVQ level 1 or 2, SCOTVEC Module, City and Guilds Craft or equivalent; Other school qualifications not already mentioned (including foreign qualifications) |  |
| Medium | Level 2 & Level 3 qualifications: SCE Higher Grade, Higher, Advanced Higher, CSYS, A Level, AS Level, Advanced Senior Certificate or equivalent; GSVQ Advanced, SVQ level 3, ONC, OND, SCOTVEC National Diploma, City and Guilds Advanced Craft or equivalent; HNC, HND, SVQ level 4 or equivalent; Other post-school but pre-Higher Education qualifications not already mentioned (including foreign qualifications) |  |
| High | Level 4 qualifications: Degree, Postgraduate qualifications, Masters, PhD, SVQ level 5 or equivalent; Professional qualifications (for example, teaching, nursing, accountancy); Other Higher Education qualifications not already mentioned (including foreign qualifications) |  |
| **Social Grade** | | |
| AB | Higher and intermediate managerial, administrative and professional occupations | Census 2011 |
| C1 | Supervisory, clerical, and junior managerial, administrative and professional occupations |  |
| C2 | Skilled manual occupations |  |
| DE | Semi-skilled and unskilled manual occupations, unemployed and lowest grade occupations |  |
| **Economic activity** |  |  |
| Employed | Economically active: In employment (part-time, full-time) | Census 2011 |
| Retired | Economically inactive: Retired |  |
| Out of labour | Economically inactive: Student; Looking after home or family; Long-term sick or disabled; Other |  |
| Unemployed | Economically active: Unemployed (Seeking work and available to start  in 2 weeks or waiting to start a job already obtained) |  |
| **Household tenure** | | |
| Owned | Owned: owns outright, owns with a mortgage or loan, shared ownership | Census 2011 |
| Private rented | Private rented: private landlord or letting agency, employer of a household member, relative or friend of a household member, other. |  |
| Social rented | Rented from council, rented from Housing association/ Registered social landlord |  |
| Live rent free | Lives rent free |  |
| **Income deprivation** | | |
| Q1 | The most deprived | SIMD 2012 |
| Q2 |  |  |
| Q3 |  |  |
| Q4 |  |  |
| Q5 | The least deprived |  |
| **Urban-rural settlements^1^** | | |
| Urban areas | Large Urban Areas - Settlements of 125,000 people and over.  Other Urban Areas - Settlements of 10,000 to 124,999 people. | Urban-rural classification in 2013-2014 |
| Small towns | Accessible Small Towns - Settlements of 3,000 to 9,999 people, and within a 30-minute drive time of a Settlement of 10,000 or more.  Remote Small Towns - Settlements of 3,000 to 9,999 people, and with a drive time of over 30 minutes to a Settlement of 10,000 or more. |  |
| Rural areas | Accessible Rural Areas - Areas with a population of less than 3,000 people, and within a 30-minute drive time of a Settlement of 10,000 or more.  Remote Rural Areas - Areas with a population of less than 3,000 people, and with a drive time of over 30 minutes to a Settlement of 10,000 or more. |  |

**References**

1 Scottish Government. Scottish Government Urban/Rural Classification 2013 - 2014. 2014 November 2014.

# **5.** **Sensitivity test for models with varying adjustments**


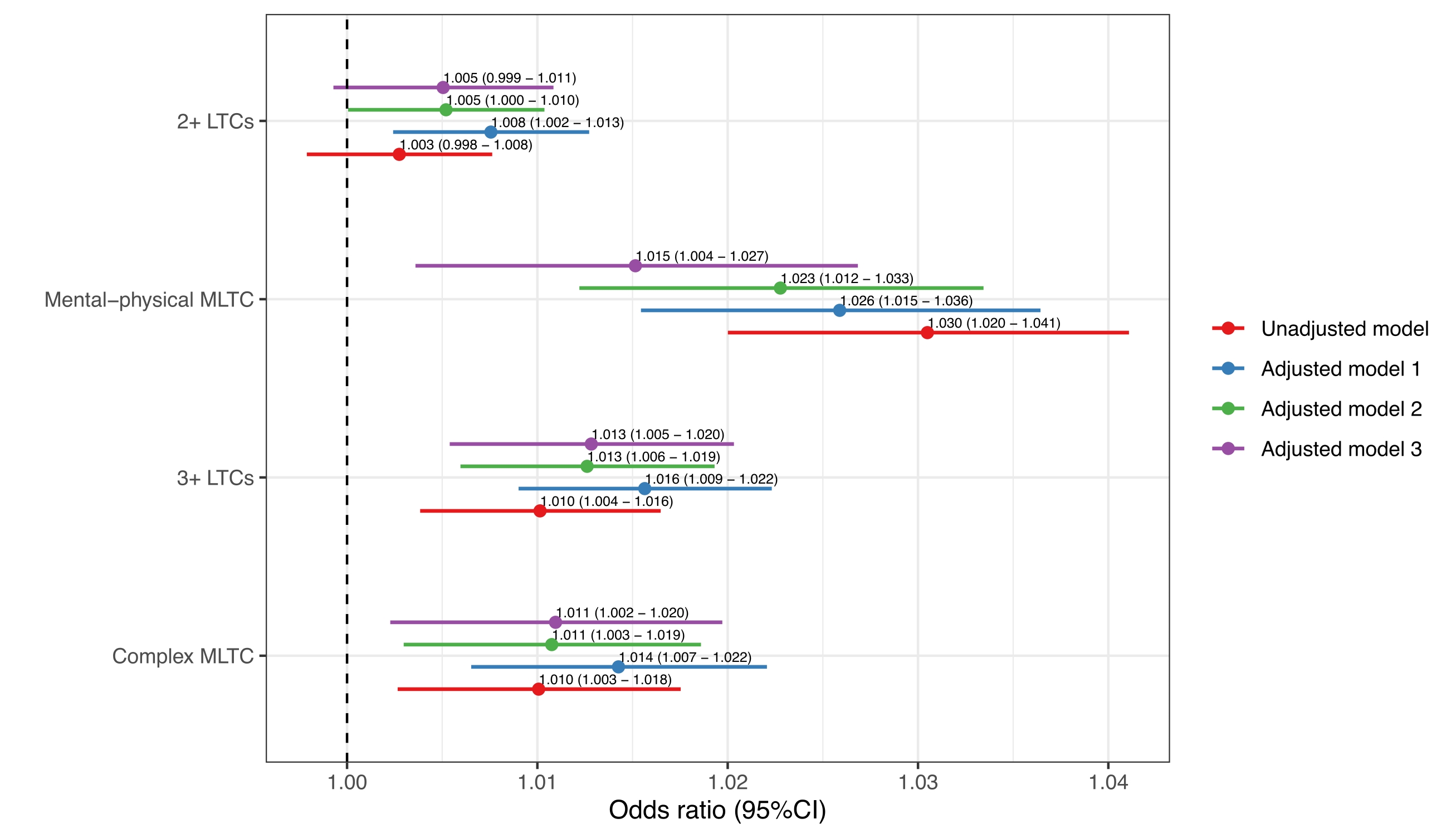


## **Figure S2.** The main effect of SGI on MLTCs under four different measurements (source: Scottish Longitudinal Study)

- Unadjusted model: the model that only controlled for SGI
- Adjusted model 1: unadjusted model + individual- and household-level covariates
- Adjusted model 2: adjusted model 1 + area-level income deprivation
- Adjusted model 3: adjusted model 2 + types of urban-rural settlement (3-fold)

**Note:** Adjusted model 3 is also the model that is presented in the main text for estimating the main effect of SGI on MLTC across different measurements.

## **Table S12**. Regression outputs from the main effect of SGI (source: Scottish Longitudinal Study)

|  | **Unadjusted model** | | **Adjusted model 1** | | **Adjusted model 2** | | **Adjusted model 3** | |
| --- | --- | --- | --- | --- | --- | --- | --- | --- |
|  | OR | 95%CI | OR | 95%CI | OR | 95%CI | OR | 95%CI |
| **‘2+ LTCs’** | 1.003 | (0.998, 1.008) | 1.008 | (1.002, 1.013) | 1.005 | (1.000, 1.010) | 1.005 | (0.999, 1.011) |
| ICC | 0.025 | (0.020, 0.031) | 0.012 | (0.007, 0.019) | 0.009 | (0.004, 0.017) | 0.008 | (0.004, 0.017) |
| AIC | 95035.97 |  | 77975.56 |  | 77820.38 |  | 77819.16 |  |
|  |  |  |  |  |  |  |  |  |
| **‘Mental-physical MLTC’** | 1.030 | (1.020, 1.041) | 1.026 | (1.015, 1.036) | 1.023 | (1.012, 1.033) | 1.015 | (1.004, 1.027) |
| ICC | 0.057 | (0.039, 0.081) | 0.014 | (0.003, 0.062) | 0.012 | (0.002, 0.067) | 0.012 | (0.002, 0.069) |
| AIC | 29470.71 |  | 26014.89 |  | 25981.45 |  | 25975.33 |  |
|  |  |  |  |  |  |  |  |  |
| **‘3+ LTCs’** | 1.010 | (1.004, 1.016) | 1.016 | (1.009, 1.022) | 1.013 | (1.006, 1.019) | 1.013 | (1.005, 1.020) |
| ICC | 0.034 | (0.026, 0.043) | 0.015 | (0.008, 0.028) | 0.011 | (0.005, 0.026) | 0.011 | (0.005, 0.026) |
| AIC | 63970.83 |  | 51104.43 |  | 50975.98 |  | 50977.59 |  |
|  |  |  |  |  |  |  |  |  |
| **‘Complex MLTC’** | 1.010 | (1.003, 1.018) | 1.014 | (1.007, 1.022) | 1.011 | (1.003, 1.019) | 1.011 | (1.002, 1.020) |
| ICC | 0.044 | (0.034, 0.057) | 0.023 | (0.014, 0.040) | 0.019 | (0.010, 0.037) | 0.019 | (0.010, 0.037) |
| AIC | 49635.37 |  | 39835.39 |  | 39738.59 |  | 39742.1 |  |

# **6. Distribution of the SGI across types of urban-rural settlement**


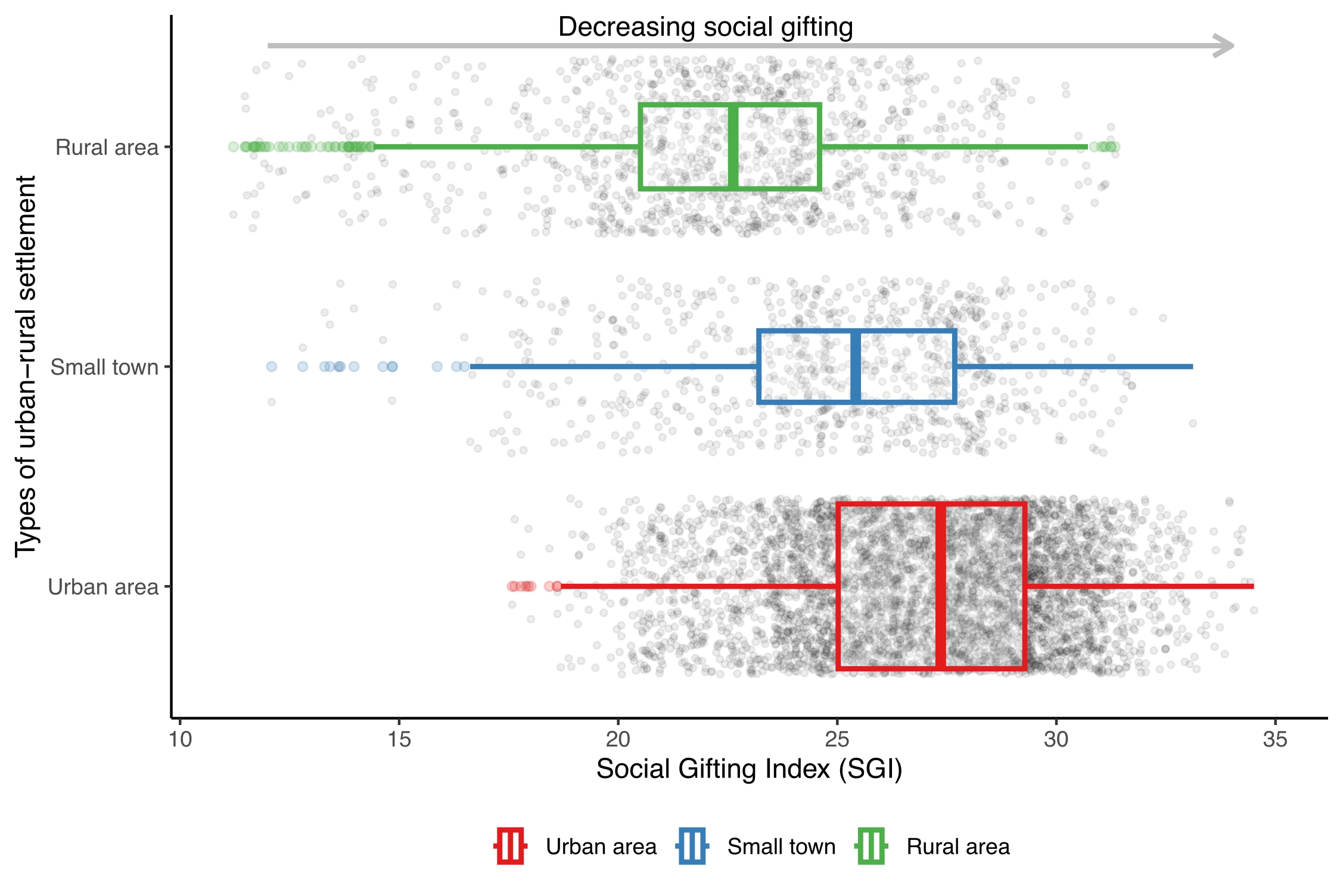


## **Figure S3**. The Distribution of SGI across types of urban-rural settlement
